# Trimming the Degrees of Freedom via a K^+^ Flux Rectifier for Safe and Long-Life Potassium-Ion Batteries

**DOI:** 10.1007/s40820-023-01178-3

**Published:** 2023-08-18

**Authors:** Xianhui Yi, Apparao M. Rao, Jiang Zhou, Bingan Lu

**Affiliations:** 1 School of Physics and Electronics, Hunan University, Changsha, 410082 People’s Republic of China; 2 Department of Physics and Astronomy, Clemson Nanomaterials Institute, Clemson University, Clemson, SC 29634 USA; 3 School of Materials Science and Engineering, Central South University, Changsha, 410083 People’s Republic of China; 4 State Key Laboratory of Advanced Design and Manufacturing for Vehicle Body, Hunan University, Changsha, 410082 People’s Republic of China

**Keywords:** Electrolytes, Degrees of freedom, Safe, Coulombic efficiency, Potassium-ion batteries

## Abstract

High degrees of freedom (DOF) for K^+^ movement in the electrolytes is desirable, because the resulting high ionic conductivity helps improve potassium-ion batteries, yet requiring support from highly free and flammable organic solvent molecules, seriously affecting battery safety. Here, we develop a K^+^ flux rectifier to trim K ion’s DOF to 1 and improve electrochemical properties. Although the ionic conductivity is compromised in the K^+^ flux rectifier, the overall electrochemical performance of PIBs was improved. An oxidation stability improvement from 4.0 to 5.9 V was realized, and the formation of dendrites and the dissolution of organic cathodes were inhibited. Consequently, the K||K cells continuously cycled over 3,700 h; K||Cu cells operated stably over 800 cycles with the Coulombic efficiency exceeding 99%; and K||graphite cells exhibited high-capacity retention over 74.7% after 1,500 cycles. Moreover, the 3,4,9,10-perylenetetracarboxylic diimide organic cathodes operated for more than 2,100 cycles and reached year-scale-cycling time. We fabricated a 2.18 Ah pouch cell with no significant capacity fading observed after 100 cycles.

**Supplementary Information:**

The online version contains supplementary material available at 10.1007/s40820-023-01178-3.

## Highlights

- High Coulombic efficiency of over 99% for dendrite-free K||Cu cell after 820 cycles.
- Year-scale-cycling performance of organic PTCDI cathode over 2,100 cycles.
- Flexible device demonstration such as fibre cell still could operate when cut into three fibre cells.

**Supplementary Information:**

The online version contains supplementary material available at 10.1007/s40820-023-01178-3.

## Graphical abstract

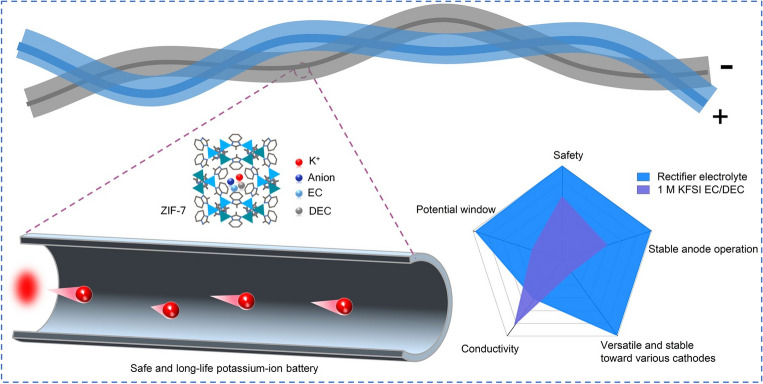

## Introduction

The current potassium-ion batteries (PIBs) are usually based on the movement of ions in the electrolyte for charging or discharging processes. The ion movement is usually three-dimensional, disordered, and require the support from highly free organic solvents molecules such as ethers and carbonate esters [1–3], which are always flammable and volatile that will seriously affect the safety in terms of thermal runaway and fire [3, 4]. The oxidative decomposition of the organic solvent molecules and anions at high voltages have always hampered the pursuit of the applicability at high voltage of some imide-potassium salt-based electrolytes [5–9]. Moreover, although the highly free organic solvent molecules ensure ion movement, they are often plagued by serious side reactions, which impede the improvement of the Coulombic efficiency [10–12]. Additionally, the electrode advancements, especially for K metal anode, appealing an enabling electrolyte to combat dendrites growth and irreversible reactions during long-term charge/discharge cycling [13–16]. The above criteria undoubtedly increase the enormous challenges in electrolytes development.

**Fig. 1:**
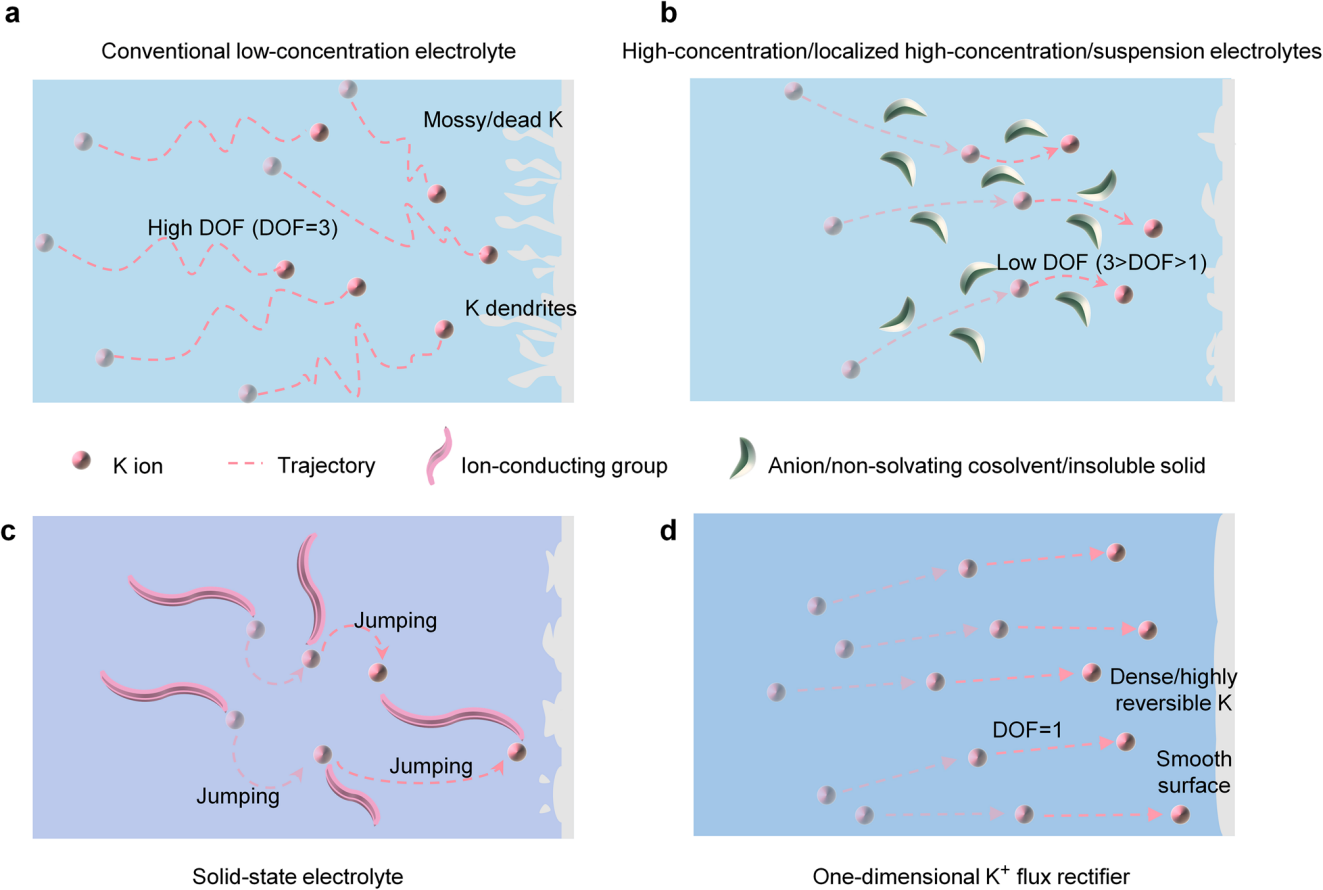
Electrolyte design strategies from the perspective of degrees of freedom (DOF). **a, b** With conventional low-concentration electrolyte (**a**), the free solvents molecules possess high DOF (DOF = 3) for K^+^ ion movement, which often leads to the formation of mossy K, dead K, K dendrites, severe side effects, and low Coulombic efficiency. For convenience, the rotational DOF for the K^+^ flux in conventional liquid electrolytes (such as 1 M KFSI EC/DEC) are omitted because they do not significantly impact ion movement. The behaviour of adding large amounts of salts/non-solvating co-solvent/or insoluble solid to the electrolytes to construct a high-concentration/localized high-concentration/suspension electrolytes, etc. as shown in (**b**), can be seen as compressing the movable space of K^+^ flux from a DOF perspective, thereby hindering the high DOF movement of K^+^ flux, forcing a decrease in its DOF, and often reducing the formation of dendrites and side effects. **c** The movement of K^+^ flux in solid-state electrolyte is mostly supported by ion-conducting groups. Although their DOF is not high and may reduce the possibility of dendrites formation, the ion jumping movement often losses much ionic conductivity. **d** K^+^ flux rectifier electrolyte described in this study enables low DOF movement of K^+^ flux (DOF = 1), decreasing electrode–electrolyte interface reactions and achieving highly stable electrochemical reactions, while also facilitating the formation of dense and uniform K metal deposition

Designing electrolytes from the perspective of degrees of freedom (DOF) allows for new and simpler explanations for many electrolyte modifications. The electrolyte system containing three-dimensional movement of K^+^ and highly free solvents mentioned above can be considered as DOF of K^+^ flux to 3, which is common in low-concentration electrolytes (Fig. 1a) [6, 17]. In recent years, many efforts have been made in develop improved electrolytes. High-concentration/localized high-concentration/suspension electrolytes have been formulated by adding large amounts of salts/inert non-solvating diluents/insoluble solids to realize some desired properties of improved battery performance (Fig. 1b) [18–21]. Based on the DOF concept, these electrolyte modifications essentially reduce the DOF of the K^+^ to promote the electrochemical property. Specifically, adding salts increased the interaction between anions and cations, thereby binding the DOF of K^+^. The salt addition also promoted the concentration of K^+^, decreasing the spatial DOF that each K^+^ can possess. The inert non-solvating diluents and insoluble solids in electrolyte systems occupy spatial positions but do not participate in the K^+^ solvation. Instead, they compress the movable space of K^+^ and decrease their spatial DOF. In addition, removing free solvents is an advisable method commonly used in the design of solid-state electrolytes (Fig. 1c) [22–24]. Nevertheless, this method may lead to significant polarization and disappointing ionic conductivity, as the loss of solvent results in low K^+^ mobility and some K^+^ may only jumping between the certain functional groups [23–26]. In advanced electrolyte designs, the solvents that are ionic lubricants in the electrolyte system are more beneficial. Notwithstanding these advances, avoiding ion jumping, maintaining the original ion mobility, and utilizing suitable amounts of salt and solvent molecules for superior battery performance is a highly demanding problem that requires great effort.

Here, we innovated an electrolyte with one-dimensional nanochannels that trim the K^+^ flux from its originally disordered movement (DOF = 3) to the one-dimensional movement (DOF = 1, Fig. 1d), while avoiding the significant loss of ionic conductivity caused by changing the form of K^+^ movement (such as jumping). Henceforth, we refer to this design scheme as the K^+^ flux rectifier electrolyte, which facilitates high safety, oxidative stability, and cycling stability of PIBs. Specifically, K||K and K||Cu cells realized thousands of cycling times with Coulombic efficiency exceeding 99%. A cycling life of 1,500 cycles with capacity retention rate of 74.7% was revealed in K||graphite cell. For the soluble organic cathode (3,4,9,10-perylenetetracarboxylic diimide, PTCDI), the cycle life was extended to 2,100 cycles. The graphite||PTCDI full cell based on our strategy also realized high performance in various aspects (an average capacity decay rate of 0.034% per cycle over 1,000 cycles; Coulombic efficiency over 99%). We also fabricated a 2.18 Ah pouch cell with small capacity change after 100 cycles. The K^+^ flux rectifier electrolyte also enabled the manufacturing of flexible fibre cells, which functioned even after being cut into three pieces. This study presents design high-performance electrolyte strategy from an all-new DOF standpoint.

## Experimental Section

### Materials

All chemicals, including vanadium pentoxide (V_2_O_5_, 99.99%), ammonium phosphate monobasic (NH_4_H_2_PO_4_, 99%), potassium fluoride (KF, 99%), oxalic acid dihydrate (H_2_C_2_O_4_·2H_2_O, 99.8%), 3,4,9,10-perylenetetracarboxylic diimide (PTCDI, 99%), commercial graphite, 1,3-diazaindene (99%), zinc acetate dihydrate (Zn(CH_3_COO)_2_·2H_2_O, 98%), potassium bis(fluorosulfonyl)imide (KFSI, 99.5%), ethylene carbonate (EC, > 99%), diethyl carbonate (DEC, > 99%), N-methyl-2-pyrrolidone (NMP, 99.5%), polyvinylidene fluoride (PVDF, average M_w_ ~ 534,000) and methanol (99.95%), ethanol (99.95%) were bought from Sigma-Aldrich. All the chemicals were used as received. 1 M KFSI EC/DEC (1/1, *v/v*) electrolyte was prepared in conventional ways. Generally, 5 mL EC (70 °C) and 5 mL DEC (28 °C) liquids were mixed quickly, then 10 mmol KFSI powder was added to the mixture solvent with constant stirring. Then the obtained 1 M KFSI EC/DEC was then divided into two parts for further usage. One part of the prepared 1 M KFSI EC/DEC solution was used as a baseline electrolyte with high K^+^ flux degrees of freedom (DOF = 3). The reactivated ZIF-7 plates and corresponding covered electrodes were immersed into the other part of 1 M KFSI EC/DEC electrolyte, and treated at 80 °C for 24 h to soak the electrolyte conductive matrix. Then, the films were taken out, wiped with tissues and dried under a vacuum for 20 h at 80 °C to get rid of any possible electrolyte solvents on the surface and hence lower the K^+^ flux DOF to a one-dimensional state (DOF = 1). As for the saturated KFSI EC/DEC electrolyte, enough KFSI powder was added to 10 ml EC/DEC (1:1, *v/v*) mixture until slight precipitate appeared.

### Materials Synthesis and Films Preparation

ZIF-7 powders were synthesized by a solution method: 1,3-diazaindene (354.4 mg, 3 mmol) and Zn(CH_3_COO)_2_·2H_2_O (263.4 mg, 1.2 mmol) were dissolved in water (100 mL) and stirred for 3 h at 28 °C. The mixture was aged for one day, and the ZIF-7 precipitates were collected by centrifugation. After immersion in 60 °C ethanol for a week and during the period, the ethanol solvent was replaced 3 times. The solid was separated by centrifugation and dried to yield the product as a white powder (ca. 450 mg). In addition, a vacuum heat treatment at 200 °C for 24 h was necessary to activate the ZIF-7 materials. Then, mixing the prepared 200 °C activated ZIF-7 materials and PVDF with a weight ratio of 9:1 in NMP to harvest the slurries. The slurries were coated on aluminum foils using a doctor blade and dried at 80 °C for 8 h, and then immersed in room temperature methanol for 30 min to obtain flexible films. The ZIF-7 slurry above was also poured onto the electrodes to obtain ZIF-7-covered electrodes. The prepared films were punched into small discs (14 mm in diameter), reactivated overnight under vacuum at 200 °C and transferred to a standard glove box.

Preparation of potassium vanadium fluorophosphates (KVPO_4_F; KVPF) [27]: Topically, the stoichiometric amounts of V_2_O_5_, KF, NH_4_H_2_PO_4_ and H_2_C_2_O_4_·2H_2_O (100% excess) were mixed and ball milled at 450 r min^−1^ for 24 h with the support of ethanol. After vacuum drying the milling slurry at 120 °C for 24 h, the material was heated at 310 °C for 2 h and 700 °C for 8 h under an argon atmosphere to obtain the final product.

Graphite, ketjen black, and PVDF (ratio: 8:1:1, in weight) were stirred in NMP for 12 h and then slurried on Al foil. After drying, punch the covered Al foil into small electrode discs weighing approximately 1.2 mg cm^−2^. In addition, KVPF, KI and PTCDI were used as active materials, and the same method (with the weight ratio changed to 7:2:1) was used to prepare electrode discs. The active materials on each piece weighed about 1.0–1.2 mg (PTCDI, KI) and 1.4–1.7 mg (KVPF).

### Electrochemical Measurements

All CR2032 coin cells were assembled in an argon-filled environment with lower than 0.5 ppm of water and oxygen, and were used to evaluate the electrochemical performance. The K foil was directly placed on the prepared K^+^ flux rectifier electrolyte-covered electrode, without adding any liquid electrolyte, to assemble the rechargeable half-cell. In addition, 1 M KFSI EC/DEC electrolyte, ca. 60 µL mg^−1^ (based on the weight of active material), and the Whatman glass fibre (separators) were also used and assembled in typical way to construct cells for comparisons. Before each electrochemical test, the batteries were left in an open circuit at room temperature overnight. For the full coin cells assembling, the pre-potassiated graphite anode was obtained by cycling for 5 cycles and then discharged to 0.01 V with the N/P ratio is about 1.05. Pouch cells were assembled by careful sandwich K^+^ flux rectifier electrolyte between the anode and cathode (double-sided integrating, N/P ratio, 1.1), without using any liquid electrolyte, here the mass loading of PTCDI to ca. 9.4 mg cm^−2^. Aluminium and nickel strips were attached as electrode tabs to the sides of the cathodes and anodes. Note that we use Al foils instead of Cu foils as current collectors for graphite anodes in order to pursue lower weight in this work. The nickel welding here is only for better and more intuitive distinction between cathode and anode after sealing. And then the cells were packed using the aluminium laminated materials with side and top sealing, followed by vacuum sealing with gentle pressing. The galvanostatic charge/discharge processes of as-prepared batteries were performed by Neware battery testing system. The Linear sweep voltammetry (LSV, with Al as the working electrode and K as counter and reference electrodes) were performed using electrochemical workstation.

### Calculation Method

Molecular dynamics (MD) simulation consisted of ZIF-7, K^−^, FSI^−^, EC, and DEC. The atomistic structures of ZIF-7 were constructed as described in Ref. [28]. The ZIF-7 characteristics, such as gravimetric and volumetric surface areas, pore-volume, and mean pore size, were calculated using Zeo^++^ software 8. The charge distribution on the ZIF-7 unit, FSI^−^, EC, and DEC molecules were obtained by exchange–correlation function and were optimized using the generalized gradient approximation. The ZIF-7 with 210 K^+^, 210 FSI^−^, 143 EC, and 78 DEC were loaded into a rectangular box with the size of *X* (27.0 Å), *Y* (27.0 Å), and *Z* (124.0 Å) by *Packmol*. Next, the Amber 2020 software was used for the molecular dynamics simulation. After minimizing, heating, and balancing the system, we embarked on the phase simulation. The minimization process was carried out under the *NVE* ensemble. In this ensemble, the number of particles* N*, the volume *V*, and the energy *E* were kept constant. The energy minimization was carried out in a total of 20,000 steps. The steepest descent method was used for the first 5,000 steps, and the conjugate gradient method was converted after 6,000 steps; the system imposes periodic boundaries (ntb = 1). Note that the energy information was outputted to *mdout* and *mdinfo* files every 100 steps. After the production phase simulation was over, the *cpptraj* module in the Amber 2020 software was used to analyse the density distribution of anions, cations, and solvent molecules in the pores of the molecular dynamic trajectory. The heating, equilibrium, and production phase process was carried out under the *NVT* (in which the temperature *T* rather than the energy *E* was fixed) using the customized MD software GROMACS. The heating process had a total of 40,000 steps with a step length of 0.5 fs; the system imposed a constant volume periodic boundary (ntb = 1), and we did not control the pressure (ntp = 0). The cutoff distance for non-bond interaction was 4.0 Å. The parameters (ntc = 2, ntf = 2) were imposed to SHAKE key length constraint, and at the same time, the pseudo-random seed was added for the Langevin thermostat. The initial temperature was 0 K, which was slowly raised to 301 K within 30,000 steps and maintained at 10,000 steps. The charging dynamics were ascertained within a temperature range of 300–400 K due to the large impact of temperature on the dynamic properties and capacity. In addition, more than 20 million steps were carried out in the production phase simulation process, and a potential field was added in the *Z* direction. We took the molecular/ion coordinates in the channel as the object and performed cluster analysis on the trajectory to obtain a snapshot of the distribution of representative channel molecules or ions, and then performed quantitative calculations of single-point energy and properties.

### Characterizations

X-ray diffraction (XRD) and *in situ* XRD measurements were performed on a Bruker D8 Advanced diffractometer. Scanning electron microscopy (SEM, JEOL JSM-6380LV FESEM) was used to characterize the morphology of all the samples. Raman spectroscopy was performed with a 533 nm diode laser, and the scattering signal was enhanced by Raman spectroscopy (SHINERS) techniques. K||K cells were assembled in a modified Swagelok cell with X-ray transparent polymer-based housing in a glovebox for X-ray computed microtomography (XCT) experiments. XCT was then carried out on diondo d2 (120 kV). The Swagelok cells were rotated 180° under the X-ray, and the shadows cast by the samples were converted to image stacks with ~ 1,800 images in each stack. The stacks were re-sliced with Tomviz software to obtain the cross-sectional and bottom surface tomography slices. Topically, the electrolytes were placed between two stainless steel plates to prepare the cells and measure their ionic conductivities [29, 30]. Note that we measured the overall ionic conductivity of the 1 M KFSI EC/DEC electrolyte-infiltrated glass fibre separator. It is more in line with the actual battery operation and more suitable for comparison. On the other hand, the prepared K^+^ flux rectifier electrolyte film was also sandwiched between two stainless steel plates. All the cells were equilibrated at room temperature for 3 h before performing the measurement. The ionic conductivity was measured by the electrochemical impedance spectroscopy (EIS) method using an electrochemical workstation in the frequency range from 0.01 Hz to 1 MHz and calculated according to equation: σ = *l/SR*. Where σ is the ion conductivity, *l* is the distance (cm) between the two stainless steel plates, *S* is the area (cm^2^) of the stainless steel plate and the *R* (Ω) was calculated from the impedance Nyquist plot.

## Results and Discussion

### Design, Properties, and Theoretical Study K^+^ Flux Rectifier Electrolyte

**Fig. 2:**
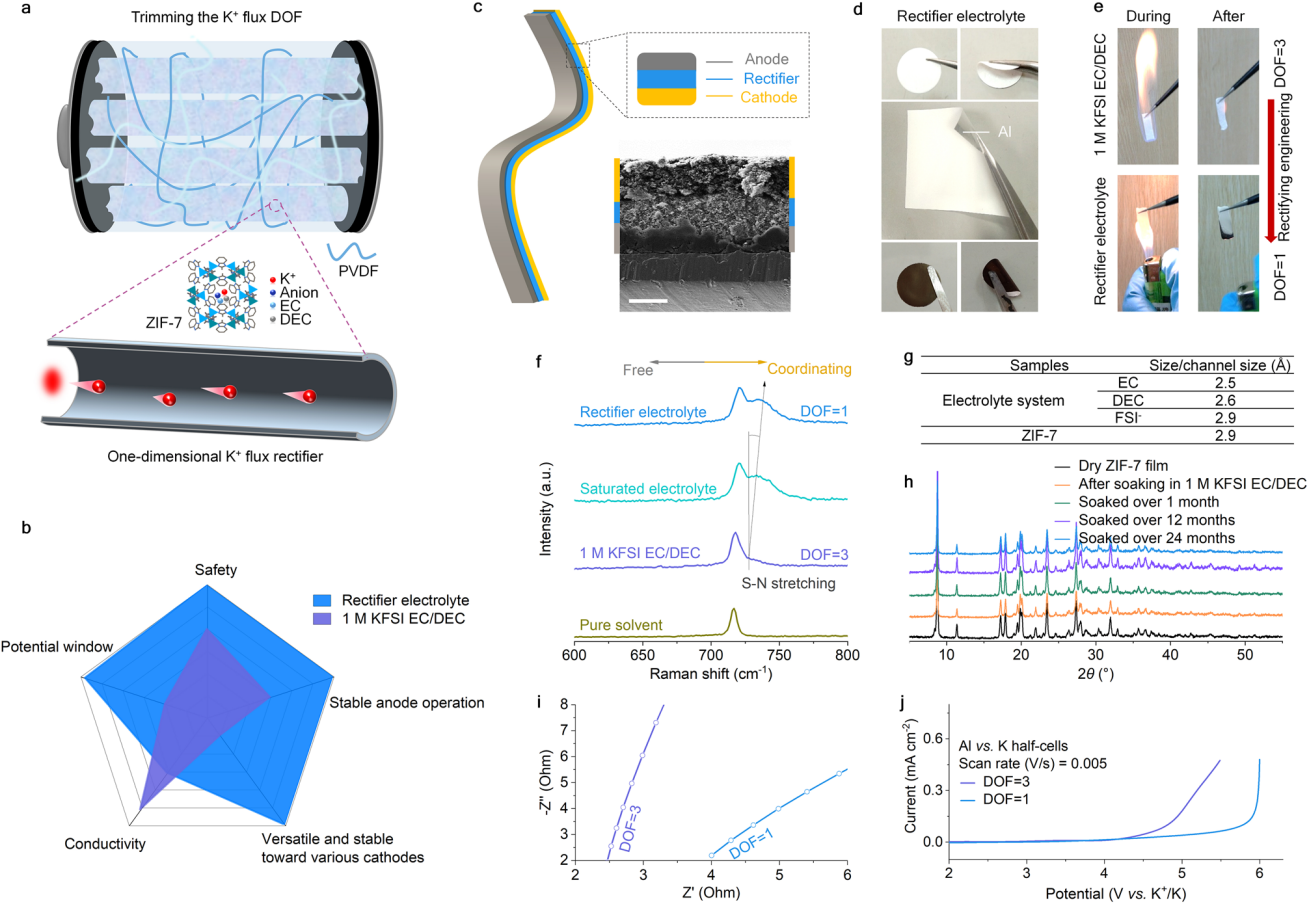
Properties of the tamed K^+^ flux rectifier. **a** Schematic illustration of the designed electrolyte for the PIBs, in which the movement of K^+^ is restricted to one-dimension. The DOF for K^+^ flux is low (DOF = 1) compared to a DOF = 3 in conventional 1 M KFSI EC/DEC electrolyte. **b** Radar chart compares key properties enhanced by the K^+^ flux rectifier electrolyte over 1 M KFSI EC/DEC electrolyte. **c** Schematic diagram of a flexible cell with the K^+^ flux rectifier electrolyte and its cross-sectional SEM image. **d** Optical images of K^+^ flux rectifiers with different shapes and sizes. **e** Comparison of the flame tests of the two electrolytes. For the 1 M KFSI EC/DEC electrolyte, a soaked Whatman glass fibre separator was used in the flame test. **f** Raman spectra for pure organic solvent (EC/DEC, 1/1, by *vol.*), 1 M KFSI EC/DEC electrolyte, saturated KFSI EC/DEC electrolyte and the K^+^ flux rectifier electrolyte. **g** Molecular size of the solvents and FSI^−^ in the electrolyte, and the channel size of the K^+^ flux rectifier. **h** Time-dependent XRD patterns of a ZIF-7 film soaked in 1 M KFSI EC/DEC electrolyte for over 24 months. **i** EIS spectra of the two electrolytes. **j** LSV tests with the two electrolytes

This work used a natural porous ZIF-7 to construct the K^+^ flux rectifier electrolyte. As schematically shown in Fig. 2a, the K^+^ flux in 1 M potassium bis(fluorosulfonyl)imide in ethylene carbonate/diethyl carbonate (1/1, by *vol.*, denoted as 1 M KFSI EC/DEC,) is restricted to one-dimensional movement (DOF = 1, Fig. S1). Compared with conventional 1 M KFSI EC/DEC electrolytes, the benefits of K^+^ flux with low DOF are as follows. Low DOF electrolyte does not require too many solvent molecules, reducing the possibility of solvent leakage and improving its safety. Thanks to the reduction of solvent molecules and the nanochannel configuration, the concentration of K^+^ is relatively increased, resulting in an increased voltage window. The K^+^ flux rectifier electrolyte is also beneficial to electrode operation, despite some loss in ionic conductivity (Fig. 2b).

The battery with a composite cathode, anode and K^+^ flux rectifier electrolyte was constructed and showed good flexibility and integration (Fig. 2c, d), laying a foundation for large-scale industrialized batteries. In addition, the flame test of the K + flux rectifier electrolyte proved meritorious than the conventional 1 M KFSI EC/DEC electrolyte. The latter was highly volatile and flammable, immediately catching fire upon ignition and burning even after removing the fire torch (Fig. 2e) [31]. But the K^+^ flux rectifier electrolyte could not be ignited because of its zero-self-extinguishing nature, resulting only in a non-fire carbonization of the ZIF-7 block due to ignition (Fig. 2e). Furthermore, when the batteries with 1 M KFSI EC/DEC electrolyte are sheared, the liquid electrolyte leaks out (Fig. S2), posing safety concerns [32]. In striking contrast, the K^+^ flux rectifier electrolyte does not pose safety concerns, thus expanding our designed strategy's applicability to open-ended battery systems, e.g., alkali metal-gas (air/O_2_/CO_2_) batteries.

The Raman characterization of the K^+^ flux rectifier and the different-concentrations KFSI EC/DEC electrolytes are shown in Fig. 2f. For pure EC/DEC hybrid solvents, the peak at 718 cm^−1^ has been assigned to the C = O bending of free molecules present in the EC/DEC hybrid solvent (Fig. 2f) [3]. The S–N stretching band of FSI^−^ (at 729 cm^−1^) appeared after 1 molar KFSI was added to the pure solvent [33]. With the continued addition of the salt powder to form a saturated KFSI EC/DEC solution, a new Raman peak located at about 735 cm^−1^ emerged. This peak is also assigned to S–N stretching, indicating its ionic configuration from a free state turned to a relatively coordinated state [33, 34]. In the case of the K^+^ flux rectifier electrolyte, the Raman signature for the ionic configuration continued to shift in a more coordinated direction, indicating that its salt concentration is even higher than the KFSI EC/DEC saturated state. The special situation may be related to our size selection (Fig. 2g). We considered the narrowest part of the solvent molecule and the solute ion, and found that it ranges from 2.5 to 2.9 Å. Therefore, we choose ZIF-7 with a channel size of 2.9 Å to store the conductive matrix, and its channel size is exactly equal to the narrowest point of FSI^−^. What’s more, we demonstrated that ZIF-7 film can stably exist in the 1 M KFSI EC/DEC electrolyte, maintaining its morphology (Fig. S3) and crystallinity (Fig. 2h) for over 24 months, confirming a good stability. This nature lays the footstone of K^+^ flux rectifier electrolyte as a stable electrolyte in the PIBs operating environment.

We used molecular dynamics (MD) simulations based on a model wherein the anions and solvent molecules were subjected to different electric fields to gain insights into the K^+^ transport mechanism (Tables S1-S2 and Figs. S4-S5) [35]. The charge distribution of each atom on different molecules (ZIF-7 unit, FSI^−^, EC, and DEC) is shown in Figs. S6-S9, which may cause the conductive matrix molecular to rotate. Our MD simulations found that K^+^ are mainly located in the centre of the traps, and anions are distributed on the surface, showing a layered morphology at 0 V (Figs. S10-S11). When an electric field of -1 or 1 V is applied, unlike at 0 V, the FSI^−^ sometimes disappears in the two-dimensional images, which may be due to the rotation of FSI^−^. In fact, the position of FSI^−^ does not undergo significant positional shifts (cf. Fig. S11), implying that the FSI^−^ is still trapped in the framework. Conversely, the electric field causes the K^+^ to begin to line up axially [35–37], which may also be why our strategy did not cause the electrolyte to lose too much ionic conductivity (0.499 mS cm^−1^, *vs.* 0.809 mS cm^−1^ for 1 M KFSI EC/DEC electrolyte) coupled with cells. Such behaviour is relatively rare in cells with high salt concentration systems (Fig. 2i), especially considering that Raman spectroscopy indicated that our salt concentration is even higher than the saturated concentration. Note that this is the overall ionic conductivity of the battery measured through electrochemical impedance spectroscopy (EIS) and has taken into account the separator and interfaces [30, 38], which is not suitable for direct comparison with that of pure liquids electrolyte. Moreover, it is worth noting that the position of solvent molecules does not change while applying positive and negative electric fields. All solvent molecules density distributions profiles remain the same, with only slight differences in the radial number distribution (Figs. S10-S12 and Movie S1).

An extensive voltage window is of great significance for advanced electrolyte, because it determines whether the electrolyte is universal [39]. The linear sweep voltammetry (LSV) shown the cycle voltage of cathode assembled with organic liquid imide-potassium salt-based electrolytes is generally not higher than 4 V (*vs.* K^+^/K) (Fig. 2j) [39]. But significantly, this electrochemical window of K^+^ flux rectifier electrolyte has enlarged to 5.9 V (*vs.* K^+^/K), much higher than that of 1 M KFSI EC/DEC electrolyte (purple curve in Fig. 2j) and provided the basis for exploring high-voltage cathodes. Also, it means that the high-voltage oxidative stability is much improved.

### Reversibility and Stability of K Metal

**Fig. 3:**
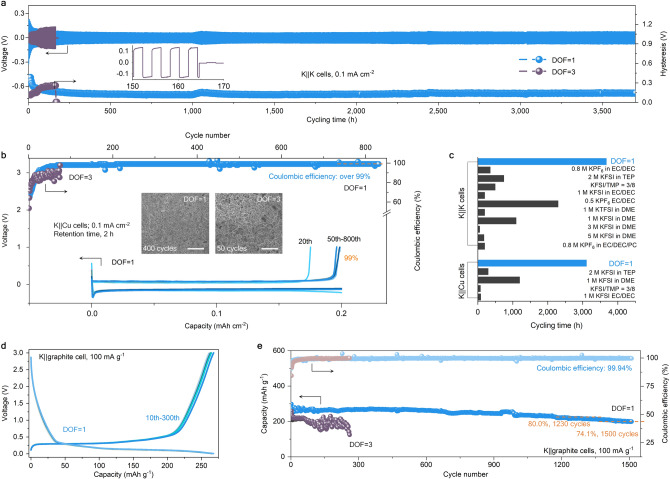
Electrochemical properties of potassium metal and a graphite anode. **a** Voltage curve and voltage hysteresis of symmetric cells with the two electrolytes. Inset: voltage data when a short circuit occurs in the DOF = 3 cell. **b** Coulombic efficiency *vs.* cycle number (top) of K||Cu cells with the two electrolytes and the selected charge–discharge profiles (bottom, voltage *vs.* capacity) of K||Cu cell cycled in K^+^ flux rectifier electrolyte (DOF = 1). Inset: SEM images of the K metal morphologies at plated states with the two electrolytes, scale bars: 10 μm. **c** Comparison of K||K and K||Cu cells running times with various electrolytes. See Table S3 and S4 for details. **d** Charge–discharge profiles of K||graphite cell. **e** Cycling performances of K||graphite cells with the two electrolytes

Using K foils and the two electrolytes (cf. Figure 2i, j), symmetric cell were assembled and used to evaluate plating-stripping behaviours (Fig. 3a). In the initial hundred hours, the polarization of the K||K cell with DOF = 3 increased and changed dramatically, with K dendrites presumably piercing the separator after ca. 160 h, leading to the cell failure. The voltage hysteresis profile increased from about 0.11 to 0.26 V during this time, mainly due to the accumulated thick passivation film caused by non-uniform K deposition and dendrite growth [40–42]. In contrast, the polarization of the K||K cell with K^+^ flux rectifier electrolyte at DOF = 1 gradually decreased and holded steady at ca. 0.06 V with a dramatically enhanced cycle life of 3,700 h. Note that the polarization increase around 1,100 h is mainly caused by a decrease in the test temperature, but fortunately, after the test temperature was recovered to room temperature, the polarization of the cell can be restored. The initial decreases in polarization may be due to battery activation and the formation of a uniform potassium surface. The reversibility of K plating/stripping was evaluated by assembling K||Cu cells (Fig. 3b). The initial Coulombic efficiency of the battery at DOF = 1 battery was 72.9%, far superior to that of DOF = 3 battery (53.5%). Next, the Coulombic efficiency with DOF = 1 battery increased and eventually reached to ~ 90%, and achieved a cycling life of 820 cycles. Such long-term cycling stability has rarely been reported in low-concentration electrolytes or high-concentration electrolytes before (Fig. 3c and Tables S3-S4), whether in K||K or K||Cu cells. Notably, even when the time of plated/stripped K was increased to 3 h, the K||Cu cell with our electrolyte still shown a high Coulombic efficiency (Fig. S13). On the contrary, the Coulombic efficiency of K||Cu with 1 M KFSI EC/DEC electrolyte dropped sharply after 80 cycles, and the cell failed completely.

Low-cost graphite is one of the most promising PIBs anode, but its long-term cycling stability needs to be improved to a thousand-cycle-scale [43–47]. A comparison of the first three hundred cycling profiles at a current density of 100 mA g^−1^ indicated that graphite’s capacity declined very quickly in the DOF = 3 battery (Fig. 3d and S14a), while it remained stable in the DOF = 1 battery. And remarkably, when using our electrolyte, the battery could deliver a stable operation for over 1,500 cycles with a Coulombic efficiency of 99.94% (Fig. 3e and S14b), outperforms the conventional DOF = 3 battery.

**Fig. 4:**
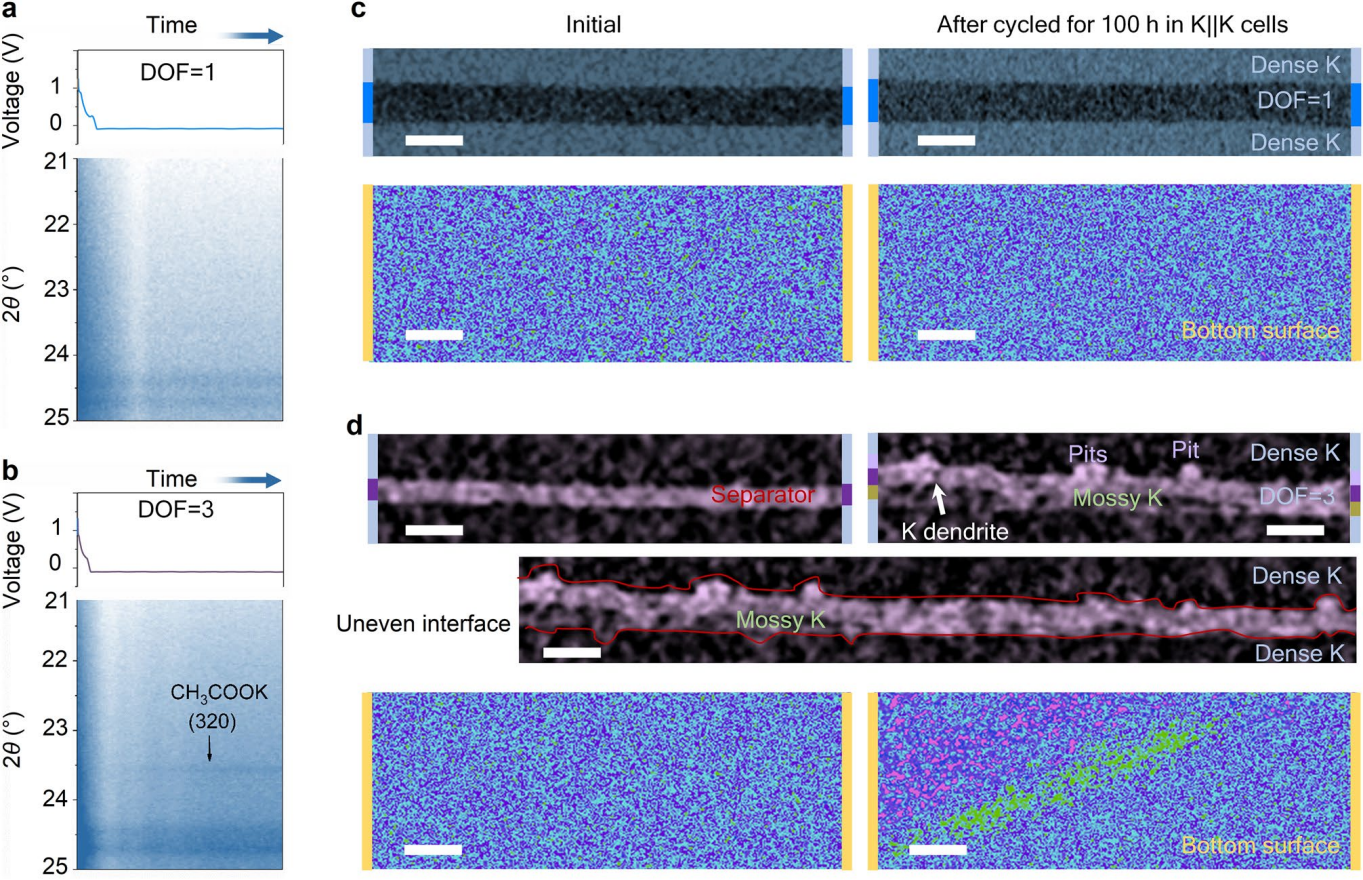
Interfacial chemical and morphological characterizations of metal electrodes. **a, b** *In situ* XRD investigations of K||Cu cells with the two electrolytes. **c** XCT images for the cross-sectional (top) and the K metal bottom surface (bottom) for visualizing both the pristine (left) and cycled (right, after 100 h) electrode−electrolyte interfaces in K||K cell configured with the K.^+^ flux rectifier electrolyte. Scale bars: 100 μm (top); 1 mm (bottom). **d** The cross-sectional and K metal bottom surface XCT images for K||K cells assembled with 1 M KFSI EC/DEC electrolyte-infiltrated glass fibre before (left) and after (right) cycling for 100 h. Scale bars: 100 μm (top); 50 μm (middle); 1 mm (bottom)

*In situ* XRD was used to study potassium deposition on the Cu current collector during the first plating process (Fig. 4a, b). The characteristic diffraction peak at ca. 23.6° is assigned to the (320) plane of potassium acetate (CH_3_COOK), which is a decomposition product of the liquid hybrid solvents (EC and DEC), revealing its poor chemical stability. For the K||Cu cell with DOF = 1, the CH_3_COOK peak is absent and the two-dimensional plot remains unchanged— the K does not have a peak in this detection area (Fig. 4b and S15), demonstrating that the solvents molecules have not decomposed—unwanted side reactions were prevented.

Non-destructive observation of the evolution of the electrolyte−electrode interface of K||K cells before and after cycling was achieved through specially designed Swagelok cells (Fig. S16) and X-ray computed microtomography (XCT). Analysis was performed before and after cycling for 100 h (0.1 mA cm^−2^, 2 h retention). For the K||K cell with DOF = 1 (Fig. 4c), K dendrites and observable interfacial changes were absent after cycling for 100 h. Also, no obvious colour changes were observed in the contact area maps across the interface, implying no change in the contact condition. On the other hand, for the K||K cell with DOF = 3, the pits on the inside surface of the cycled K electrode were evident, and mossy K (deposited low-density K) was also present (Fig. 4d and S17). A more troubling observation was the presence of potassium dendrites piercing the separator (Fig. S17), consistent with the failure of the corresponding K||K battery over ca. 160 h. In addition, the pits formed at the bottom surface would roughen the surface and decrease the contact area, increasing polarization and adversely affecting the cycling stability. Collectively, these data imply that the K^+^ flux rectifier electrolyte impedes electrolyte degradation, dendrite growth, and the onset of mossy K formation, thereby prolonging the cycle life of the K metal battery.

### Stable Operation of Cathodes

**Fig. 5:**
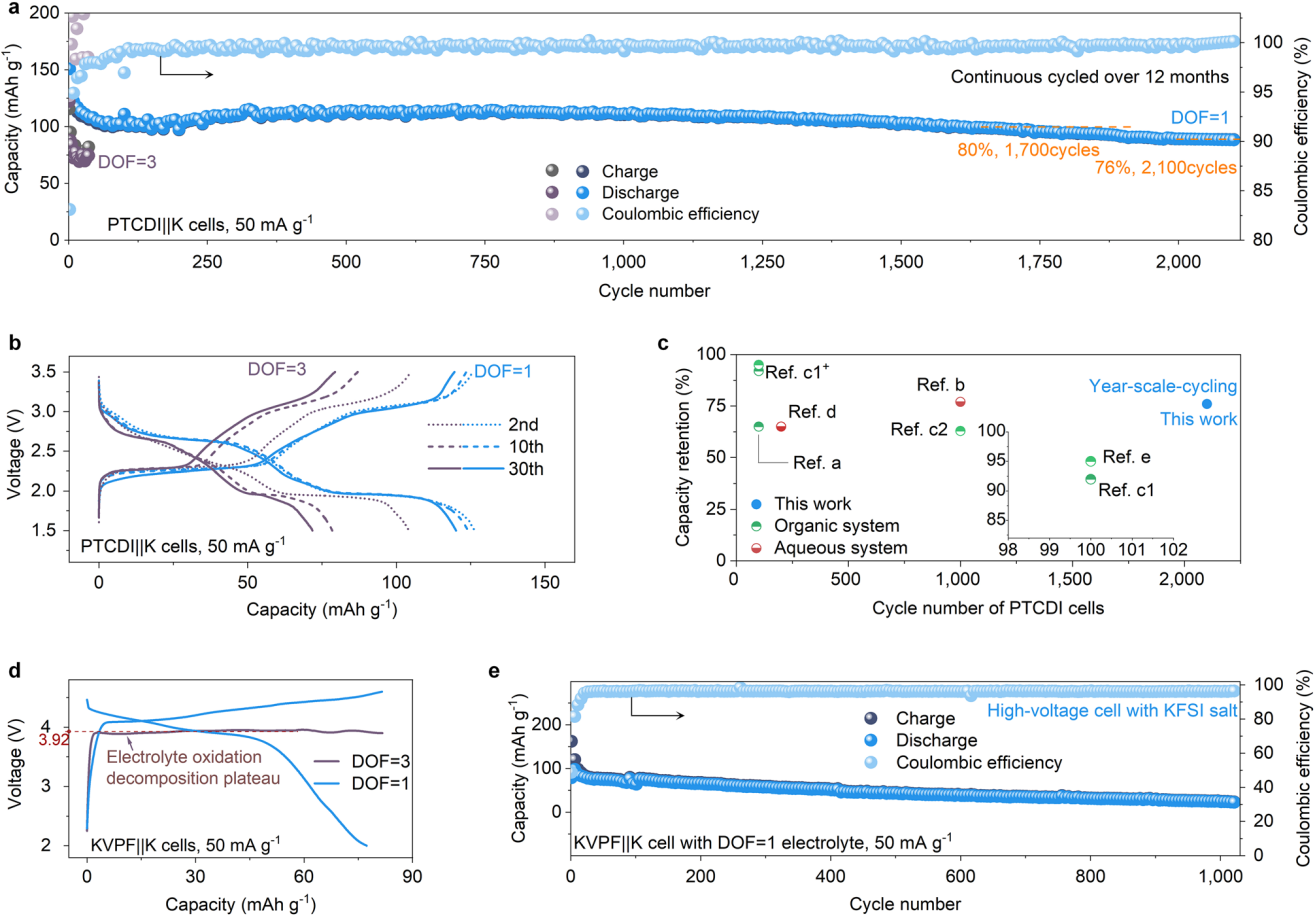
Electrochemical performances at the cathodes side. **a, b** Long-term cycling performance (**a**) and typical charge–discharge profiles (**b**) of PTCDI||K cells with the two electrolytes. **c** A comparison of the results of optimized PTCDI cells from different perspectives in reported works, including organic and aqueous systems, and electrodes optimization. See Table S5 for details. **d** Voltage profiles KVPF||K cells with the two electrolytes. **e** Cycling properties of KVPF||K cells with the DOF = 1 electrolyte

A class of materials—organic cathodes—with potentially low cost and sustainability has been pursued actively for PIBs [48–50]. However, a long-standing challenge for the practical implementation of organic cathodes (such as PTCDI) is the dissolution of electrode materials by organic solvents, which eventually results in poor cycle life (Fig. S18) [29, 51]. The PTCDI||K cell with DOF = 3 delivered a first discharge capacity of ~ 122 mAh g^−1^ at 50 mA g^−1^ (Fig. 5a). But after 30 cycles, the corresponding capacity reduced to ~ 75 mAh g^−1^, delivering capacity retention of only ~ 62% (Fig. S19). On the contrary, because the solvents molecules in the K^+^ flux rectifier electrolyte are trapped and moreover, it is impossible for potassiated PTCDI to enter the framework due to the limitation of available migration paths (Fig. S20), there is no solvent present to dissolve the PTCDI cathode [52]. Consequently, the same batch of PTCDI ran stably, maintaining a capacity retention rate of 76% after 2,100 cycles (Fig. 5a, b). Notably, the: (i) PTCDI||K cell with DOF = 1 stable operation over 2,100 cycles (over 12 months) and a Coulombic efficiency of over 99% confirmed that the cycle life of PTCDI has been greatly extended; and (ii) PTCDI cathode’s performance with our K^+^ flux rectifier electrolyte is remarkable compared to the reported works (Fig. 5c and Table S5). This excellent performance underscores the unique advantages of our electrolyte in organic electrodes (including organic anodes).

The KVPO_4_F (KVPF, Fig. S21) cathode was also used to verify the high-voltage (2.0—4.6 V) galvanostatic cycling test (Fig. 5d) [27, 53]. When the DOF = 3 battery was charged to about 4.0 V, the voltage no longer increased and a plateau was evident, indicating that side reactions have occurred (such as organic solvent decomposition), consistent with our LSV test results and the reported works [39, 54]. In contrast, the DOF = 1 battery can be charged to 4.6 V smoothly, achieving a remarkable cycling stability (over 1,000 cycles). An average output voltage as high as 3.92 V could be harvest, with a low capacity decay rate of about 0.075% per cycle from the 10th to 1,000^th^ cycle (Fig. 5e), which is the first time that high-voltage KVPF cathode has shown such electrochemical behaviour in imide-potassium salt electrolyte (Table S6). Our K^+^ flux rectifier electrolyte greatly broadens the upper voltage limit of potassium imide salts[53]. Taken together, the good operation of different electrodes types with our electrolyte confirms that the long-life K^+^ flux rectifier electrolyte is multifunctional.

### Full Cell Performance and Device Demonstration

**Fig. 6:**
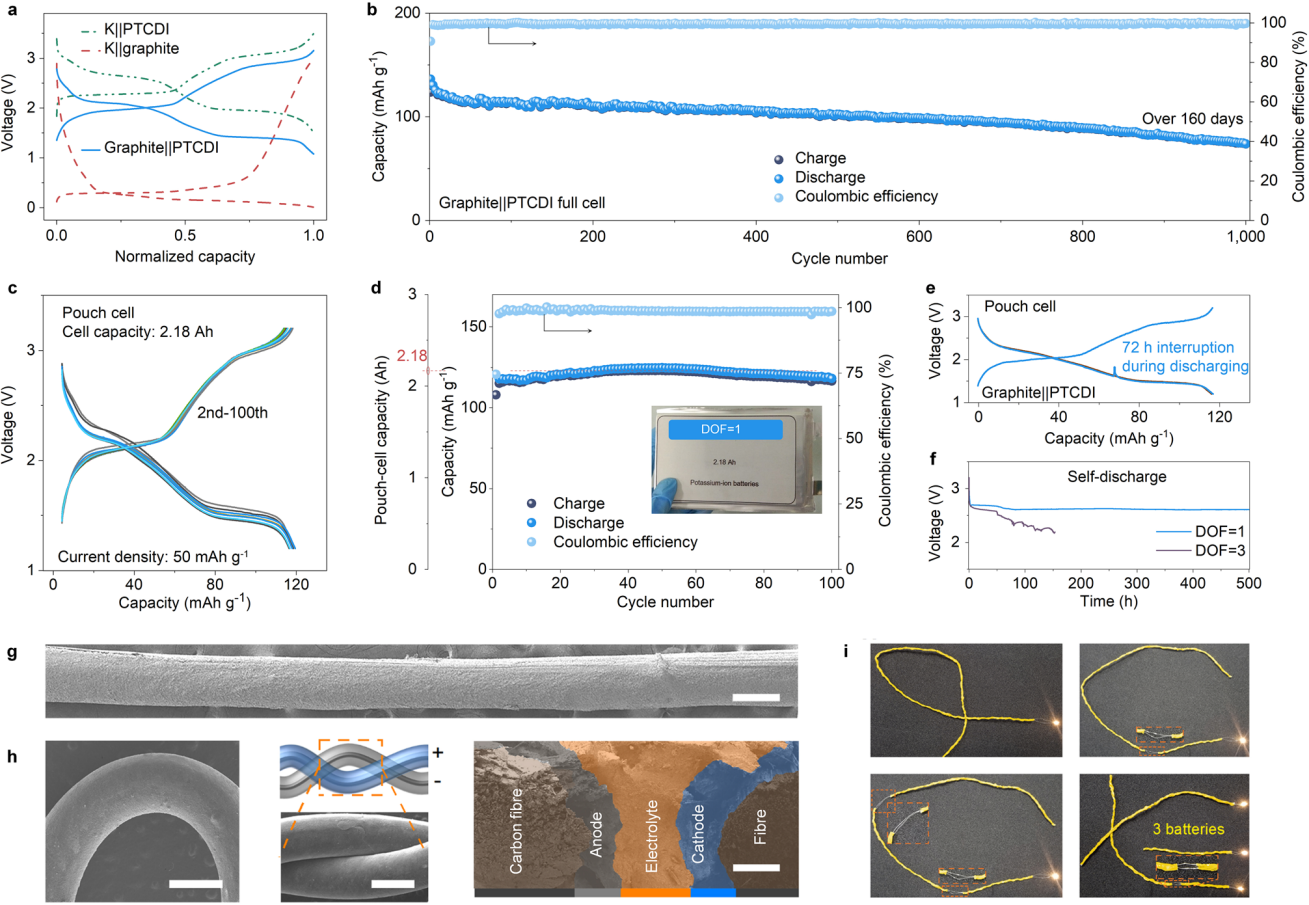
Cycling characteristics and functional extensions of full cells. **a** The typical charge–discharge profiles of the half and full cells. **b** Performances of graphite||PTCDI full cell under continuous cycling for over 160 days at a current density of 50 mA g^−1^. **c, d** Charge–discharge profiles (**c**) and cycling performance (**d**) of a 2.18 Ah pouch cell with the K^+^ flux rectifier under a high cathode mass loading (ca. 25 mg cm^−2^). Inset (**d**): an optical photograph of the fabricated pouch cell. **e** Charge–discharge profiles of graphite||PTCDI cell with a 72 h’ interruption during operation. **f** The voltage *vs*. time profiles after different cells were fully charged. **g, h** SEM images of a flexible fibre electrode with a uniform active material layer (**g**), schematic diagram of winding (**h**), and corresponding cross-sectional view (**h**). Scale bars: 100 nm (h, right), 1 mm (other). **i** The single flexible fibre cell could power a LED under different bending conditions and still operate when cut into three fibre cells

To further evaluate the application prospects of the K^+^ flux rectifier electrolyte, and considering the complexity of KVPF preparation and the instability of product quality [27, 55], we chose the commercially available PTCDI cathode to construct full cells. We have constructed a full cell that integrates PTCDI cathode and graphite anode through a simple multiple coating method, which is amenable for large-scale industrialization (Figs. 2c and 6a). This kind of cell could deliver a capacity of 123 mAh g^−1^ **(**based on the mass of the cathode**)** at a current density of 50 mA g^−1^ with the Coulombic efficiency over 99% (Fig. 6b). Moreover, an average capacity decay rate of 0.034% per cycle over 1,000 cycles was harvested. This battery exhibits high safety and good resistance to damage, e.g., it lights up the light-emitting diode screen even if it was cut twice (Fig. S22). By increasing the mass loading of PTCDI to ca. 9.4 mg cm^−2^, an areal capacity of 1.16 mAh cm^−2^ was obtained. Thus, we fabricated a 2.18 Ah pouch cell (seven-layer double-sided integrating) (Fig. 6c), which exhibited 100 cycles of stable operation with a small reduction in capacity (Fig. 6d). It should be mentioned that several key parameters listed in Table S7 can be improved further (which we could not because of the limited technology at our laboratory level) to fabricate superior pouch cells (e.g., mass loading per square centimetre) [55–57]. The stable discharge profile shown in Fig. 6e was collected after interrupting the cell cycling for 72 h, indicating excellent ion storage capacity. We further investigated the self-discharge rate of the full cells configured with the two electrolytes (Fig. 6f) [58]. The cell’s self-discharge with our electrolyte is suppressed compared to a cell assembled with the 1 M KFSI EC/DEC electrolyte-infiltrated glass fibre separator. In short, these demonstrated properties compete with industry-relevant cycling, safety, and voltage stability requirements, thus promising new solutions for a wide range of industrialized large-scale energy storage devices, such as Li-ion and Na-ion batteries (Fig. S23).

Lastly, to prove our electrolyte strategy is scalable, we also fabricated a fibre cell consisting of two carbon fibres wound together, as shown in Fig. 6g. The SEM image of the cross-section shows its internal structure. Between the two carbon fibre current collectors (black), the cathode (blue), electrolyte (orange), and anode (grey) were sequentially coated. The fibre battery has good flexibility (Fig. 6h) and can be cut into three fibre batteries of shorter lengths. Yet, these fibre batteries continued lighting the LED (Fig. 6i). Therefore, if a portion of the battery is defunct, it could be cut off from the rest to get a shorter working battery, which is very useful in some special extreme applications, such as in space where it is inconvenient to repair or replace.

## Conclusions

In this work, we proposed an electrolyte design from a new perspective of DOF and successfully developed a K^+^ flux rectifier electrolyte. The designed K^+^ flux rectifier electrolyte in which the DOF = 1 for the K^+^ flux helps overcome several drawbacks of conventional electrolytes, such as 1 M KFSI EC/DEC. PIBs with the K^+^ flux rectifier electrolyte exhibited stable, safe, and long-time cycling life (year-scale-cycling) and achieved stable cycling at a higher voltage (up to 4.6 V) than conventional electrolyte’s 4.0 V. Developing the rectifier electrolyte described in this study represents a big step towards safer and stable PIBs. We expect that the concept of designing electrolytes from DOF perspective can be widely applied to the operation of various metal-ion batteries, especially in metal-organics and open-ended alkalis-gas (air/O_2_/CO_2_) cells, because of its distinctive mechanical/electrochemical properties.

### Supplementary Information

Below is the link to the electronic supplementary material.

Supplementary file1 (MP4 32115 kb)

Supplementary file2 (PDF 1457 kb)
