# Supplementary material for: Trimming the Degrees of Freedom via a K+ Flux Rectifier for Safe and Long-Life Potassium-Ion Batteries
Source: Nanomicro Lett. 2023 Aug 18;15:200. doi: 10.1007/s40820-023-01178-3 (PMC10439096; doi:10.1007/s40820-023-01178-3)
Supplement: Supplementary file 2 — Supplementary file2 (PDF 1457 kb) [file 40820_2023_1178_MOESM2_ESM.pdf]

Supporting Information for

Trimming the Degrees of Freedom via a K^+ Flux Rectifier for Safe and Long-Life Potassium-Ion Batteries

Xianhui Yi[1], Apparao M. Rao[2], Jiang Zhou[3], and Bingan Lu[1, 4, *]

[1] School of Physics and Electronics, Hunan University, Changsha 410082, P.R. China

[2] Department of Physics and Astronomy, Clemson Nanomaterials Institute, Clemson University, Clemson, SC 29634, USA

[3] School of Materials Science and Engineering, Central South University, Changsha 410083, P.R. China

[4] State Key Laboratory of Advanced Design and Manufacturing for Vehicle Body, Hunan University, Changsha 410082, P.R. China

*Corresponding author. E-mail: luba2012@hnu.edu.cn (Bingan Lu)

Supplementary Figures and Tables

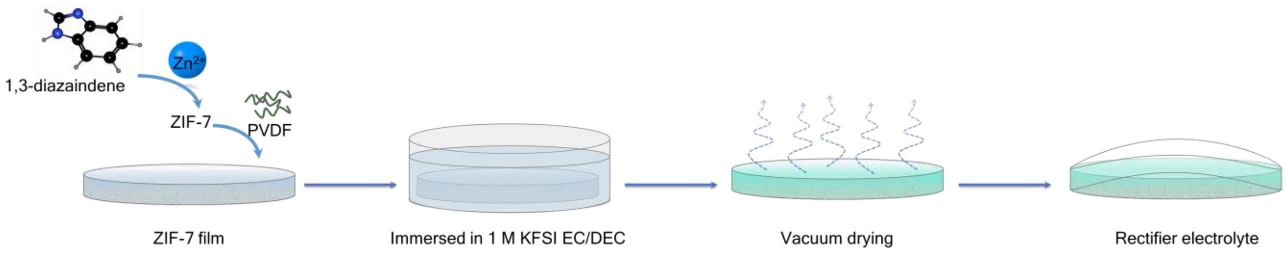

Fig. S1 Schematic illustration for preparing the rectifier electrolyte

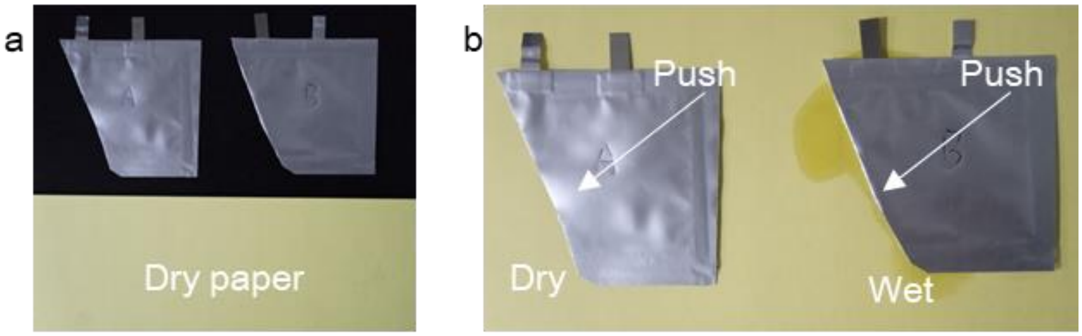

Fig. S2 The electrolyte leakage test. Comparison of electrolyte leakage in rechargeable batteries with K^+ flux rectifier electrolyte (marked A) and 1 M KFSI EC/DEC electrolyte (marked B) before test (**a**), and pressing the pouch on a piece of dry paper (**b**)

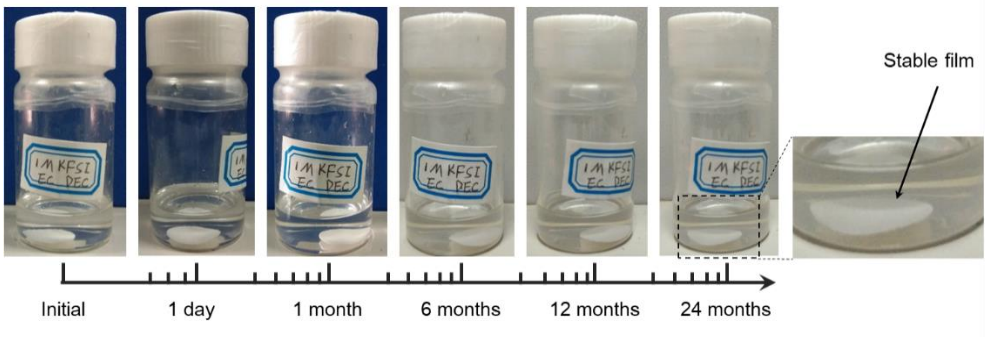

Fig. S3 Morphology stability test of ZIF-7 film. It was soaked in the electrolyte for over 2 years. It retained its stable morphology in the electrolyte

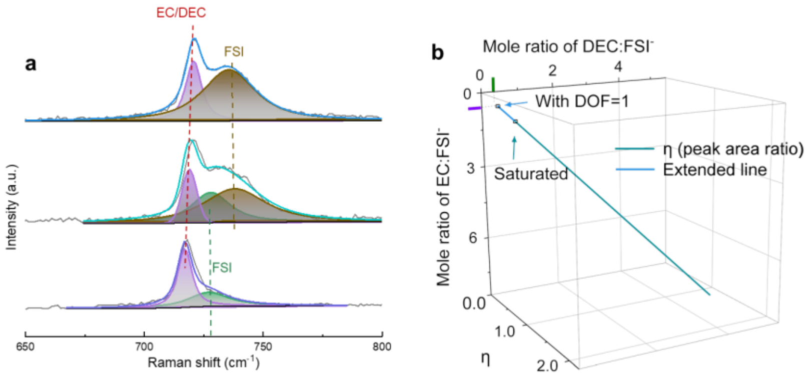

Fig. S4 Raman characterizations. **a**, Raman spectra for 1 M KFSI EC/DEC (bottom), saturated KFSI EC/DEC (middle) and K^+ flux rectifier electrolyte (top). The peaks and corresponding sources have been marked with corresponding colors. **b**, Curve of EC:FSI and DEC:FSI peak area ratio [η, η = peaks (FSI^-)/peak (EC/DEC)] in K^+ flux rectifier electrolyte (extended line). The values of purple and green labels in the figure are 0.67 and 0.37, respectively, indicating that the molar ratio in the K^+ flux rectifier electrolyte is: EC:KFSI = 0.67, and DEC:KFSI = 0.37

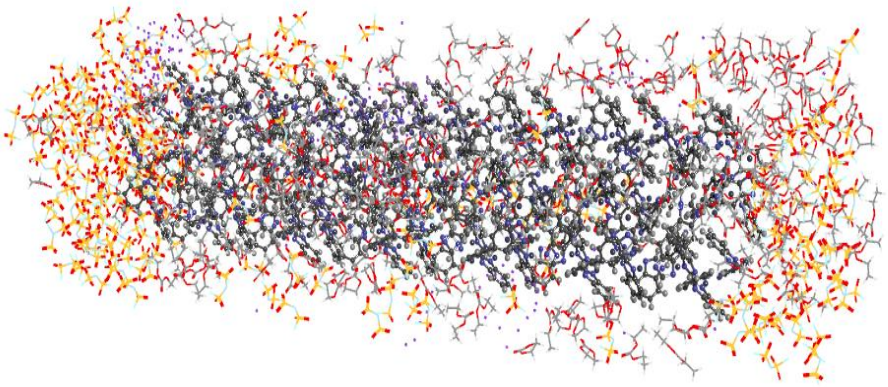

Fig. S5 MD simulation. The electrolyte system consisted of ZIF-7, K^+, FSI^-, EC, and DEC. The K^+, FSI^-, EC, and DEC ratio was 1:1:0.67:0.37

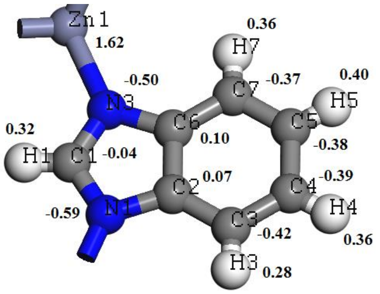

Fig. S6 Calculated charge distribution in ZIF-7 unit. The total charge is 0

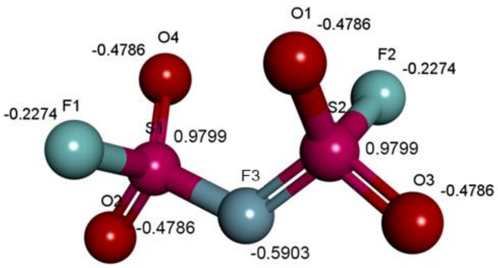

Fig. S7 Calculated charge distribution in FSI^-. The total charge is -1 e^-

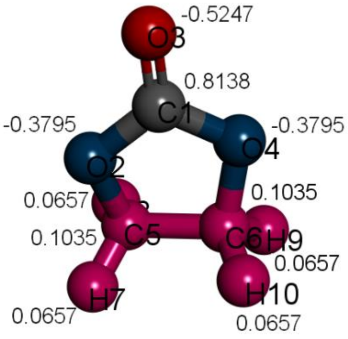

Fig. S8 Calculated charge distribution in EC. The total charge is 0

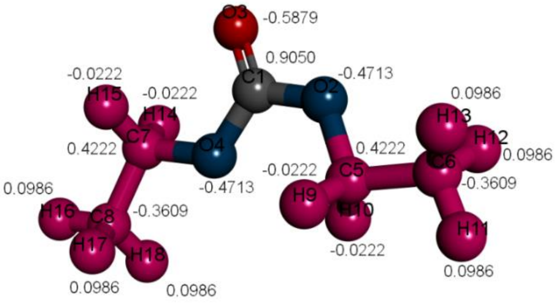

Fig. S9 Calculated charge distribution in DEC. The total charge is 0

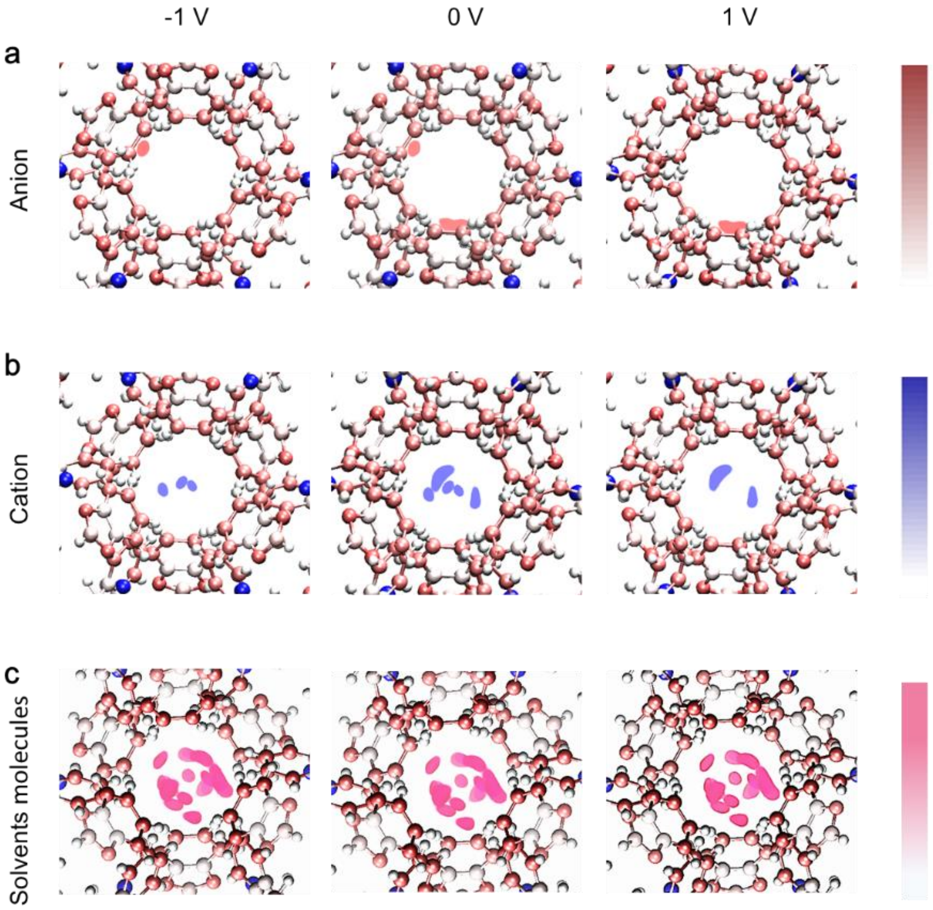

Fig. S10 Ion and molecule density distributions in the K^+ flux rectifier electrolyte. In-plane two-dimensional image of the FSI^- (**a**), K^+ (**b**), and solvents molecules (**c**) distribution in the studied traps. Each image is based on simulated data averaged along the pore axis

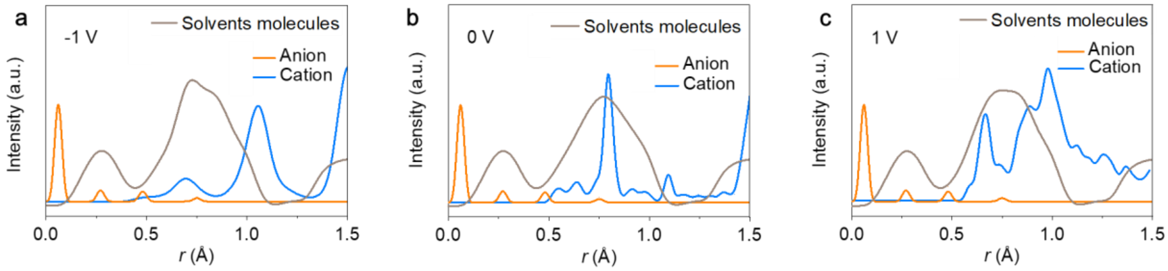

Fig. S11 Ion and molecule radial number distributions in the K^+ flux rectifier electrolyte. **a**, -1 V. **b**, 0 V. **c**, 1 V. r = 0.0 Å stands for the periphery

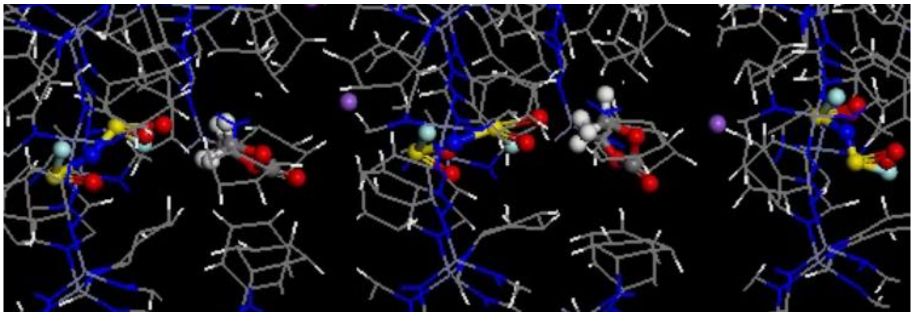

Fig. S12 A snapshot of the MD simulation at 1 V. Colors for different elements: H-white, K-purple, C-gray, O-red, N-blue, S-orange, and F-cyan

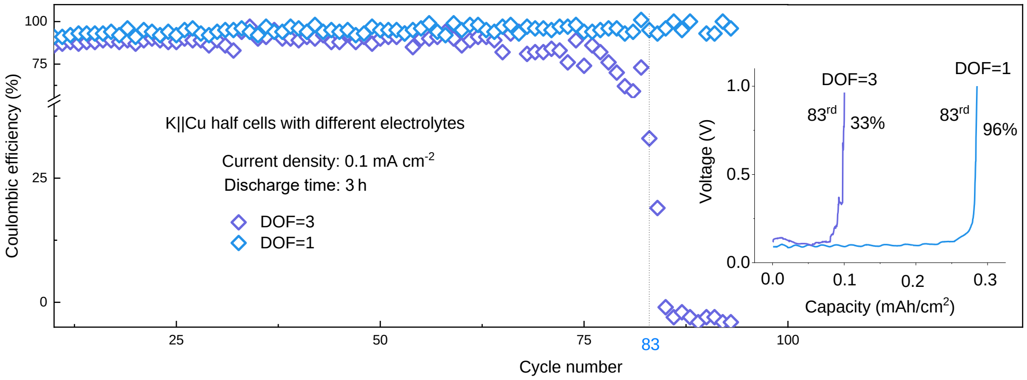

Fig. S13 Coulombic efficiency of K||Cu cells (0.1 mA cm^{-2}, 0.3 mAh cm^{-2}). Inset: the 83rd charging profiles of K||Cu cells cycled with the two electrolytes. The percentages indicate the respective Coloumbic efficiencies

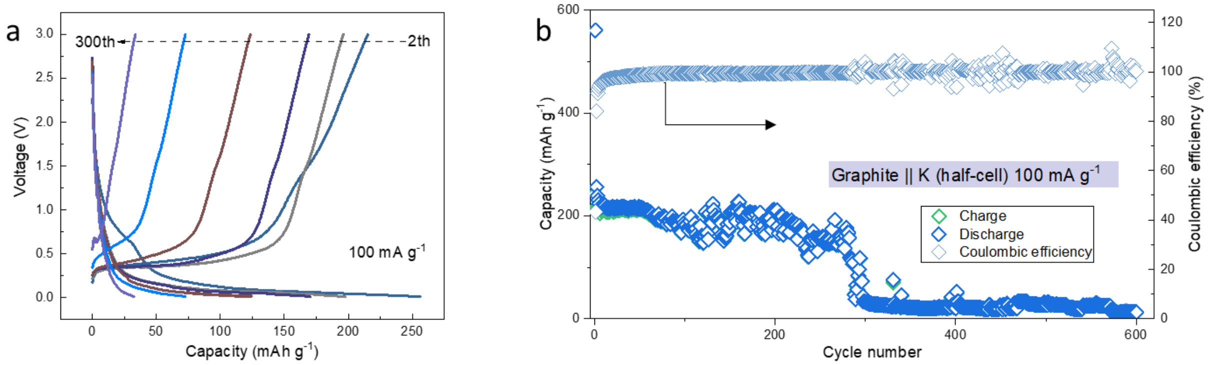

Fig. S14 Electrochemical performance of the graphite half-cell cycled in the 1 M KFSI EC/DEC electrolyte. **a**, Charge-discharge profiles in the first 300 cycles. **b**, Long-term cycle performance

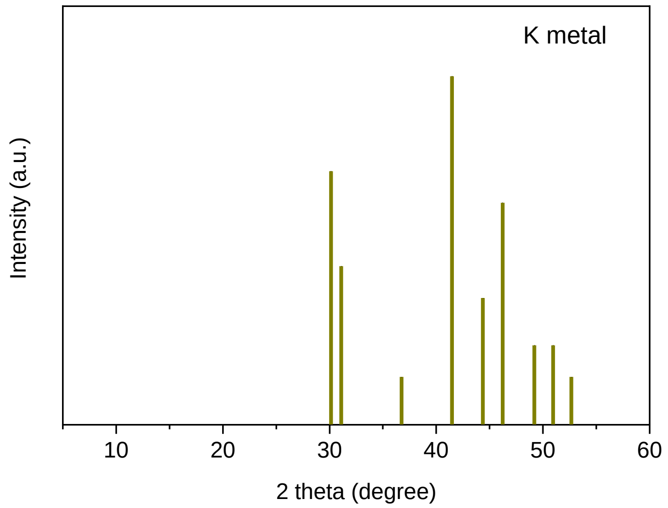

Fig. S15 XRD pattern of K metal

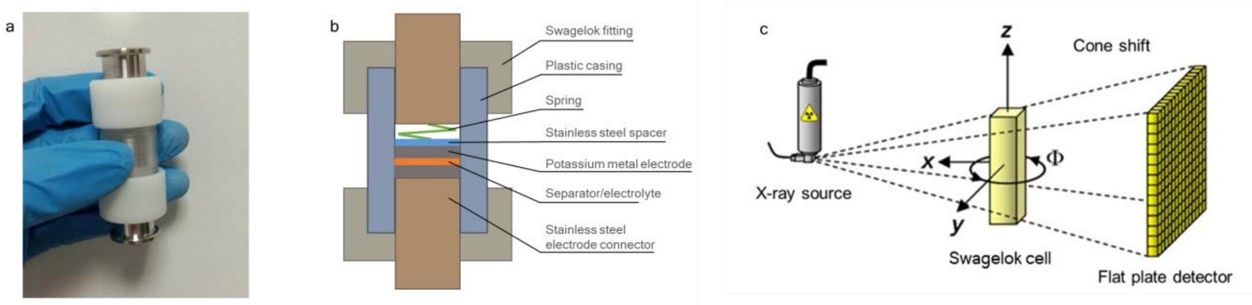

Fig. S16 Swagelok cell. **a**, Optical image. **b**, Schematic diagram of the internal structure. **c**, Schematic diagram of XCT testing

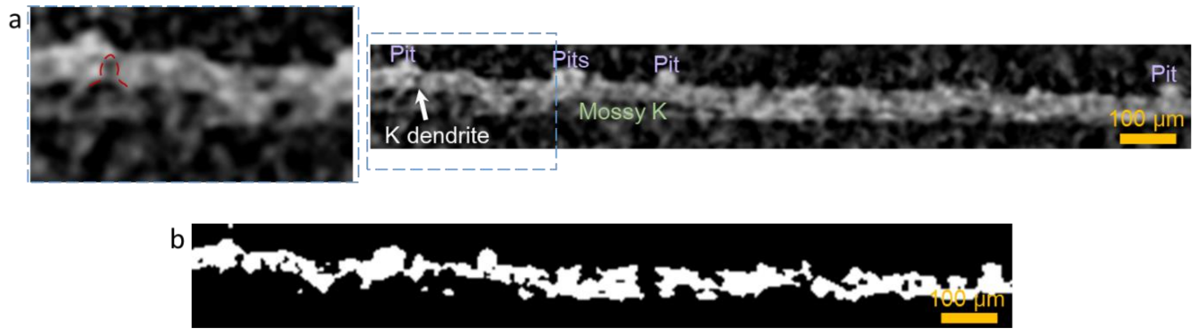

Fig. S17 Magnified XCT images. **a,** Cross-sectional analysis of synchrotron hard XCT for visualizing the cycled (0.1 mA cm^{-2}, 0.2 mAh cm^{-2}; 100 h in K||K symmetric cells) electrode-electrolyte interface with 1 M KFSI EC/DEC electrolyte. A potassium dendrite can be observed at the location marked with a red dashed line (left) and depicted with a white arrow (right). **b,** Black and white view of the electrode-electrolyte interface after cycling

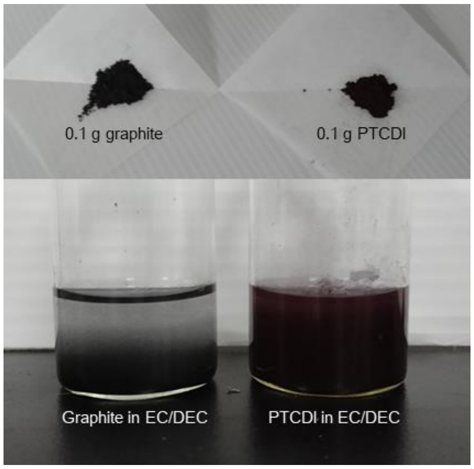

Fig. S18 Solubility test. Photos of 0.1 g graphite (for comparison) and 0.1 g PTCDI dissolved in EC/DEC (1/1, *by vol.*) solutions

If PTCDI were insoluble, the dispersion would appear clear (like 0.1 g graphite in EC/DEC), as in the case of graphite, rather than turbid.

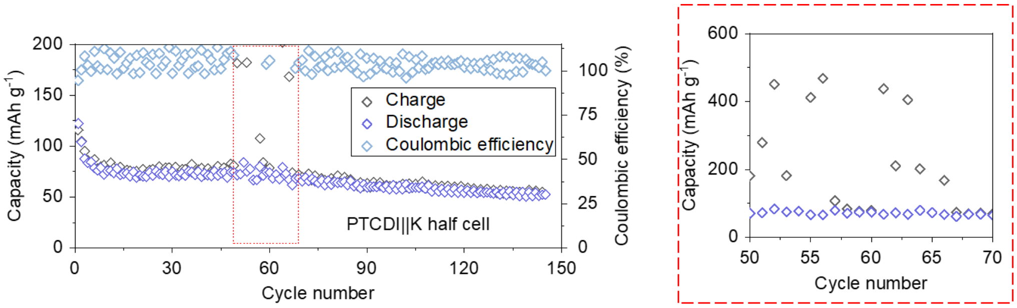

Fig. S19 Electrochemical performance of the PTCDI||K cell with 1 M KFSI EC/DEC electrolyte

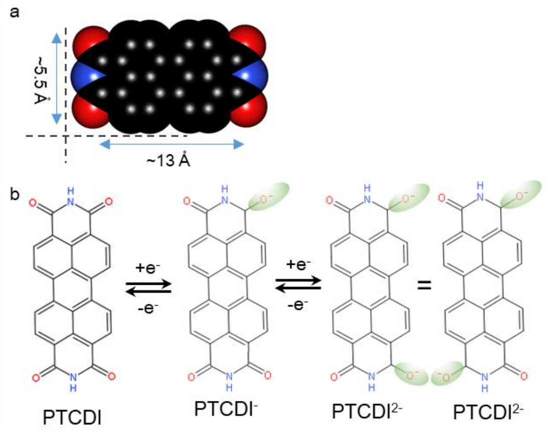

Fig. S20 The visualization of molecular valence orbitals. **a,** PTCDI, **b,** $PTCDI^-$ and $PTCDI^{2-}$. The available size in K^+ flux rectifier electrolyte for PTCD to enter is ca. 2.9 Å in width, smaller than the length of $PTCDI^{n-}$ (n = 1, 2) redox intermediates (~5.5 Å)

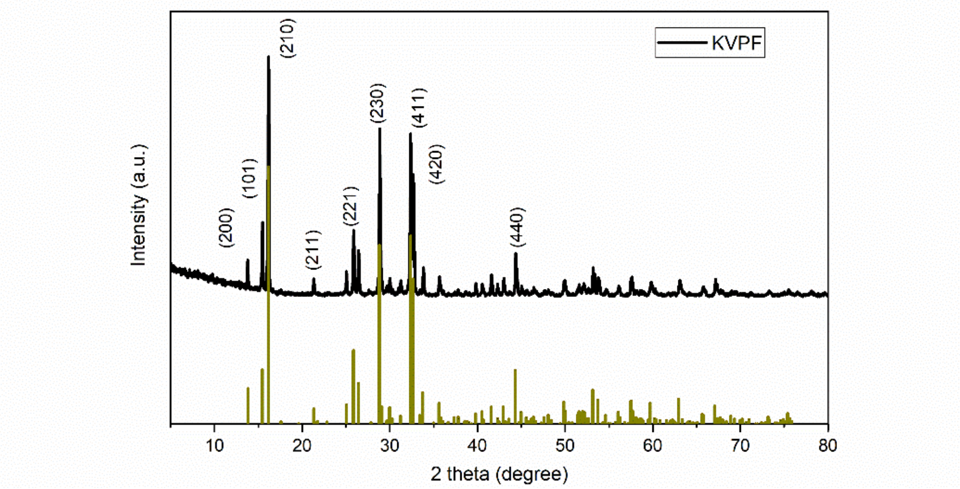

Fig. S21 The well-matched XRD pattern of KVPF powder sample

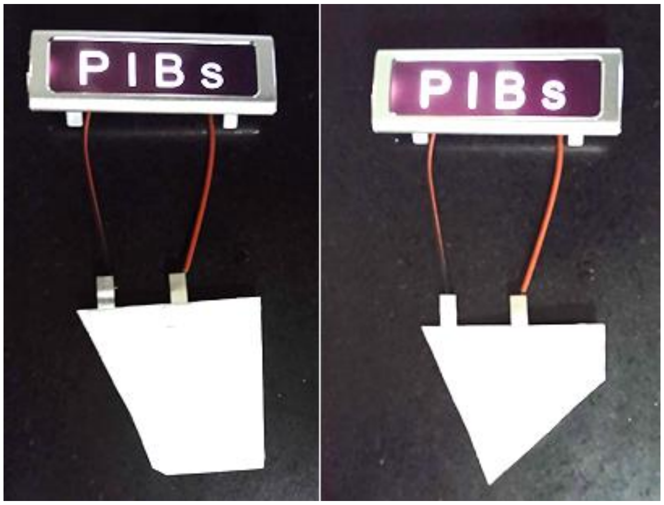

Fig. S22 Safety test. The cell with our K^+ flux rectifier electrolyte could power a LED display even after being cut twice

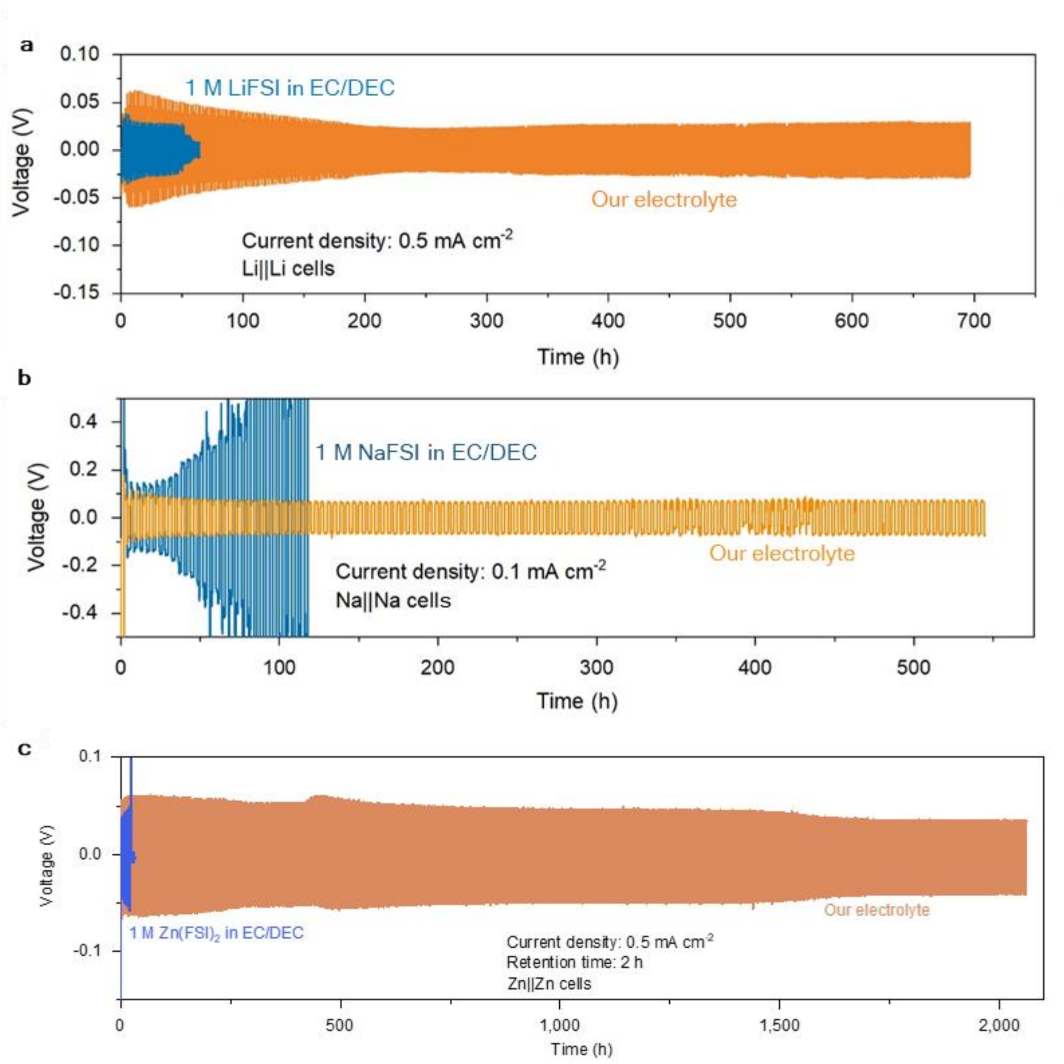

Fig. S23 Attempts of adopting our K^+ flux rectifier electrolyte strategy in various metal symmetric cells. **a,** Li||Li cells. **b,** Na||Na cells. **c,** Zn||Zn cells. The huge differences in the charge-discharge profiles for these symmetric cells cycled with different electrolytes confirm that our electrolyte strategy can prolong the cycle life of various metal batteries.

Table S1 Physical properties of ZIF-7

Surface area (m^2 g^{-1})	V (cm^3 g^{-1})	ρ (g cm^{-3})	d (nm)	l (nm)	P
2000.075	0.103	1.390	0.293	0.514	0.143

V is the pore volume normalized to mass; ρ is the mass density; *d* is the pore diameter; *l* represents the volume-to-surface rate (i.e., the pore volume divided by its surface area); *P* denotes the porosity of the ZIF-7.

Table S2 The number of cation-anion pairs, the box size used in our simulations, and the length of the modeled K^+ flux rectifier electrolyte

Number of K^+-FSI^- pairs	Box size: X, Y, Z (nm)	Length (nm)
210.0	2.7, 2.7, 12.4	9.0

Table S3 Comparison of the ability of electrolytes to maintain cycling life in K||K cells

Electrolytes	Cycling life (hours)	References
0.8 M KPF_6 in EC/DEC	~90	*Proc. Natl. Acad. Sci. USA* **117**, 5588-5594 (2020) [S1]
	360	
2 M KFSI in TEP	750	*Angew. Chem. Int. Ed.* **59**, 3638-3644 (2020) [S2]
KFSI/TMP = 3/8	500	*Adv. Mater.* **33**, 2006313 (2021) [S3]
1 M KFSI in EC/DEC (FEC, 5*wt*%)	~200	*Joule* **2**, 1534-1547 (2018) [S4]
0.5 KPF_6 EC/DEC	2,300	*Adv. Energy Mater.* **9**, 1902697 (2019) [S5]
1 M KTFSI in DME	200	*Nat. Commun.* **9**, 1339 (2018) [S6]
1 M KFSI in DME	1,100	*ACS Energy Lett.* **7**, 401-409 (2022) [S7]
3 M KFSI in DME	58	*Nano Energy* **96**, 107131 (2022) [S8]
1 M KPF_6 in DME	180	*ACS Energy Lett.* **5**, 3124-3131 (2020) [S9]
5 M KFSI in DME		
0.8 M KPF_6 in EC/DEC/PC	200	*Adv. Mater.* **32**, 1906735 (2020) [S10]
DOF=1	3,700	This work

TEP: triethyl phosphate; TMP: trimethyl phosphate; FEC: 4-fluoro-1,3-dioxolan-2-one; DME: 1,2-dimethoxyethane; PC: propylene carbonate; KTFSI: potassium bis(trifluoromethanesulfonyl)imide

Table S4 Comparison of the ability of electrolytes to maintain cycling life in K||Cu cells

Electrolytes	Cycling life (hours)	References
2 M KFSI in TEP	~300	*Angew. Chem. Int. Ed.* **59**, 3638-3644 (2020)[S2]
1 M KFSI in DME	1,200	*J. Am. Chem. Soc.* ***139**, 9475-9478* (2017)[S11]
KFSI/TMP = 3/8	80	*Adv. Mater.* **33**, 2006313 (2021)[S3]
1 M KFSI EC/DEC	90	*Angew. Chem. Int. Ed.* **59**, 3638-3644 (2020)[S2]
DOF=1	3,110	This work

TEP: triethyl phosphate; DME: 1,2-dimethoxyethane; TMP: trimethyl phosphate

Table S5 Comparison of the electrochemical performances of PTCDI-based electrodes for potassium-ion batteries with different electrolytes

Electrolytes	Labels	Electrodes	Current density	Initial capacity (mAh g^{-1})	Cycles (capacity retention)	References
1 M KPF_6 in PC/EC	Ref. a	PTCDI	500 A g^{-1}	~125	100 (65%)	*J. Mater. Chem. A* **7**, 24454-24461 (2019)[S12]
22 M KCF_3SO_3 aqueous electrolyte	Ref. b	PTCDI	20C	110	1,000 (77%)	*Nat. Energy* **4**, 495-503 (2019)[S13]
5 M KFSI in EC/DMC/DEC	Ref. c1	PTCDI	100 mA g^{-1}	156	100 (92%)	*J.Mater. Chem. A* 7, 20127-20131 (2019)[S14]
5 M KFSI in DME	Ref. c2		1,000 mA g^{-1}	157	1,000 (62.9%)	
KCF_3SO_3 modified aqueous electrolytes	Ref. d	PTCDI	1,000 mA g^{-1}	~122	200 (65%)	*Nat. Sustain.* **5**, 225-234 (2022)[S15]
1 M KPF_6 + 0.05 M in DME	Ref. e	PTCDI-DAQ	100 mA g^{-1}	220	100 (95%)	*Adv. Funct. Mater.* **30**, 2000675 (2020)[S16]
DOF=1	This work	PTCDI	50 mA g^{-1}	122	2,100 (76%)	This work

PC: propylene carbonate; DMC: dimethyl carbonate; DME: 1,2-dimethoxyethane; LiTFSI: lithium bis(trifluoromethanesulfonyl)imide

Table S6 Comparison of the electrochemical performances of KVPF||K cells with different electrolytes

Electrolytes	Electrodes	Current density	Initial capacity (mAh g^{-1})	Cycling life (cycles)	References
0.7 M **KPF_6** in EC/DEC	$KVPO_4F$	5 mA g^{-1}	~105	30	*Adv. Energy Mater.* **8**, 1801591 (2018)[S17]
1 M **KPF_6** in EC/PC/FEC	$KVPO_4F$/C	20 mA g^{-1}	106.5	100	*Nano Lett.* **22**, 4933-4940 (2022)[S18]
1 M **KPF_6** in PC	$KVPO_4F$	20 mA g^{-1}	~78	150	*Sci. Bull.* **67**, 151-160 (2022)[S19]
0.6 M **KPF_6** in PC/FEC	Multi-component coated $KVPO_4F$	20 mA g^{-1} (activation) 50 mA g^{-1} (cycling)	95	50	*Chem. Eng. J.* **433**, 134634 (2022)[S20]
0.8 M **KPF_6** in EC:DEC	$KVPO_4F$@3DC	500 mA g^{-1}	60	550	*Sci. Bull.* **65**, 1242-1251 (2020).[S21]
0.7 M **KPF_6** in EC:DEC	$KVPO_4F$	5 mA g^{-1}	68	50	*Chem. Commun.* **53**, 5208-5211 (2017)[S22]
DOF=1	$KVPO_4F$	50 mA g^{-1}	~90	1,000	This work

PC: propylene carbonate; FEC: 4-fluoro-1,3-dioxolan-2-one

Table S7 Parameters for the pouch cell with our electrolyte

Parameters	Pouch cell
Specific capacity (PTCDI) (mAh g^{-1})	123±4
Mass loading (PTCDI) (mg cm^{-2})	9.4±0.2
Cathode mass loading (mg cm^{-2})	13.4±0.3
Areal capacity (single face) (mAh cm^{-2})	1.156±0.02
Average output voltage (V)	1.72
Active area (cm^2)	(9×15)×2×7=1,890
Al foil (mg cm^{-2})	4.75 ± 0.02
Package+tabs (g)	2
Pouch-cell mass (g)	52.88
Capacity (Ah)	2.18
Energy (Wh)	3.75

Movie S1

All solvent molecules density distributions profiles remain the same, with only slight differences in the radial number distribution. Colors for different elements: H-white, K-purple, C-gray, O-red, N-blue, S-orange, and F-cyan.

Supplementary References

[S1] P. Hundekar, S. Basu, X. Fan, L. Li, A. Yoshimura et al., In situ healing of dendrites in a potassium metal battery. Proc. Natl. Acad. Sci. **117**, 5588-5594 (2020). https://doi.org/10.1073/pnas.1915470117

[S2] S. Liu, J. Mao, Q. Zhang, Z. Wang, W. Kong Pang et al., An intrinsically non-flammable electrolyte for high-performance potassium batteries. Angew. Chem. Int. Ed. **59**, 3638-3644 (2020). https://doi.org/10.1002/anie.201913174

[S3] S. Liu, J. Mao, L. Zhang, W. Kong Pang, A. Du et al., Manipulating the solvation structure of nonflammable electrolyte and interface to enable unprecedented stability of graphite anodes beyond 2 uears for safe potassium-ion batteries. Adv. Mater. **33**, 2006313 (2021). https://doi.org/10.1002/adma.202006313

[S4] W. Zhang, W. K. Pang, V. Sencadas, Z. Guo, Understanding high-energy-density Sn_4P_3 anodes for potassium-ion batteries. Joule **2**, 1534-1547 (2018). https://doi.org/10.1016/j.joule.2018.04.022

[S5] H. Wang, J. Hu, J. Dong, K. C. Lau, L. Qin et al., Artificial solid-electrolyte interphase enabled high-capacity and stable cycling potassium metal batteries. Adv. Energy Mater. **9**, 1902697 (2019). https://doi.org/10.1002/aenm.201902697

[S6] Y. Gu, W. Wang, Y. Li, Q. Wu, S. Tang et al., Designable ultra-smooth ultra-thin solid-electrolyte interphases of three alkali metal anodes. Nat. Commun. **9**, 1339 (2018). https://doi.org/10.1038/s41467-018-03466-8

[S7] J. Park, Y. Jeong, M. Hilmy Alfaruqi, Y. Liu, X. Xu et al., Stable solid electrolyte interphase for long-life potassium metal batteries. ACS Energy Lett. **7**, 401-409 (2022). https://doi.org/10.1021/acsenergylett.1c02354

[S8] J. Wang, W. Yan, J. Zhang, High area capacity and dendrite-free anode constructed by highly potassiophilic Pd/Cu current collector for low-temperature potassium metal battery. Nano Energy **96**, 107131 (2022). https://doi.org/10.1016/j.nanoen.2022.107131

[S9] J. Zhang, Z. Cao, L. Zhou, G. Park, L. Cavallo et al., Model-based design of stable electrolytes for potassium ion batteries. ACS Energy Lett. **5**, 3124-3131 (2020). https://doi.org/10.1021/acsenergylett.0c01634

[S10] P. Liu, Y. Wang, Q. Gu, J. Nanda, J. Watt et al., Dendrite-free potassium metal anodes in a carbonate electrolyte. Adv. Mater. **32**, 1906735 (2020). https://doi.org/10.1002/adma.201906735

[S11] N. Xiao, W. D. McCulloch, Y. Wu, Reversible Dendrite-Free Potassium Plating and Stripping Electrochemistry for Potassium Secondary Batteries. J. Am. Chem. Soc. **139**, 9475-9478 (2017). https://doi.org/10.1021/jacs.7b04945

[S12] Y. Bai, W, Fu, W. Chen, Z. Chen, X. Pan et al., Perylenetetracarboxylic diimide as a high-rate anode for potassium-ion batteries. J. Mater. Chem. A **7**, 24454-24461 (2019). https://doi.org/10.1039/C9TA07605K

[S13] L. Jiang, Y. Lu, C. Zhao, L. Liu, J. Zhang et al., Building aqueous K-ion batteries for energy storage. Nat. Energy **4**, 495-503 (2019). https://doi.org/10.1038/s41560-019-0388-0

[S14] M. Xiong, W. Tang, B. Cao, C. Yang, C. Fan, A small-molecule organic cathode with fast charge–discharge capability for K-ion batteries. J. Mater. Chem. A **7**, 20127-20131 (2019). https://doi.org/10.1039/C9TA06376E

[S15] J. Ge, L. Fan, A. M. Rao, J. Zhou, B. Lu, Surface-substituted Prussian blue analogue cathode for sustainable potassium-ion batteries. Nat. Sustain. **5**, 225-234 (2022). https://doi.org/10.1038/s41893-021-00810-7

[S16] Y. Hu, Y. Lu, C. Zhao, L. Liu, J. Zhang et al., Novel insoluble organic cathodes for advanced organic K-ion batteries. Adv. Funct. Mater. **30**, 2000675 (2020). https://doi.org/10.1002/adfm.202000675

[S17] H. Kim, D. Seo, M. Bianchini, R.J. Clément, H. Kim et al., A new strategy for high-voltage cathodes for K-ion batteries: Stoichiometric $KVPO_4F$. Adv. Energy Mater. **8**, 1801591 (2018). https://doi.org/10.1002/aenm.201801591

[S18] J. Liao, X. Zhang, Q. Zhang, Q. Hu, Y. Li et al., Synthesis of $KVPO_4F$/carbon porous single crystalline nanoplates for high-rate potassium-ion batteries. Nano Letters **22**, 4933-4940 (2022). https://doi.org/10.1021/acs.nanolett.2c01604

[S19] Y. He, Y. Xu, M. Zhang, J. Xu, B. Chen et al., Confining ultrafine SnS nanoparticles in hollow multichannel carbon nanofibers for boosting potassium storage properties. Sci. Bull. **67**, 151-160 (2022). https://doi.org/10.1016/j.scib.2021.09.020

[S20] X.-D. He, L.-M. Zhang, C.-H. Jiang, C.-H. Chen, Elevating cyclability of an advanced $KVPO_4F$ cathode via multi-component coating strategy for high-performance potassium-ion batteries. Chem. Eng. J. **433**, 134634 (2022). https://doi.org/10.1016/j.cej.2022.134634

[S21] Z. Liu, J. Wang, B. Lu, Plum pudding model inspired $KVPO_4F$@3DC as high-voltage and hyperstable cathode for potassium ion batteries. Sci. Bull. **65**, 1242-1251 (2020). https://doi.org/10.1016/j.scib.2020.04.010

[S22] K. Chihara; A. Katogi; K. Kubota; S. Komaba, $KVPO_4F$ and $KVOPO_4$ toward 4 volt-class potassium-ion batteries. Chem. Commun. **53**, 5208-5211 (2017). https://doi.org/10.1039/C6CC10280H
